# Linc01133 contributes to gastric cancer growth by enhancing YES1-dependent YAP1 nuclear translocation via sponging miR-145-5p

**DOI:** 10.1038/s41419-022-04500-w

**Published:** 2022-01-11

**Authors:** Yujing Sun, Yuan Tian, Junyi He, Yaru Tian, Guohao Zhang, Ruinan Zhao, Wen-jie Zhu, Peng Gao

**Affiliations:** 1 Key Laboratory for Experimental Teratology of the Ministry of Education and Department of Pathology, School of Basic Medical Science, Cheeloo College of Medicine, Shandong University, Jinan, PR China; 2 Department of Pathology, Qilu Hospital, Cheeloo College of Medicine, Shandong University, Jinan, PR China; 3 Department of Radiation Oncology, Shandong Cancer Hospital and Institute, Shandong First Medical University and Shandong Academy of Medical Science, Jinan, PR China

**Keywords:** Gastric cancer, Cell growth

## Abstract

The long intergenic non-coding RNA linc01133 is reported to be oncogenic in various malignancies. However, the role and mechanism of linc01133 in regulating gastric cancer growth is still not clear. In the present study, we found that linc01133 was significantly upregulated in gastric cancer tissues compared to non-tumorous gastric tissues. Linc01133 over-expression significantly correlated with tumor size and tumor differentiation in gastric cancer patients. The expression of linc01133 was regulated by c-Jun and c-Fos collaboratively. In both in vitro and in vivo studies, linc01133 was shown to promote gastric cancer cell growth. Linc01133 localized in the cytoplasm and functioned as an endogenous competing RNA of miR-145-5p to upregulate the expression of YES1, which was proved to be the target gene of miR-145-5p. By promoting YES1-dependent YAP1 nuclear translocation, linc01133 upregulated the expression of the key cell cycle regulators CDK4, CDK6 and cyclin D1 to promote G1-S phase transition. Thus, our study unveiled the function and mechanism of linc01133 regulating cell cycle progression in gastric cancer.

## Introduction

Gastric cancer (GC) is one of the leading causes of malignancy-associated deaths. Although much has been improved in disease diagnosis and treatment, the prognosis of patients with GC in advanced stages remains non-optimistic. Thus, it’s important to elucidate the molecular mechanisms underlying GC tumorigenesis and progression.

Recently, transcriptomic studies reveal that the majority of the transcriptional events is to produce noncoding RNAs, among which miRNAs and long noncoding RNAs are drawing increasing attention [1]. Long noncoding RNAs (lncRNAs) are a group of RNAs more than 200nt in length with no protein-coding potential. It has been reported that lncRNAs could interact with DNAs, miRNAs or proteins, participating in a variety of biological and pathological cellular processes via regulating target gene expression at transcriptional, post-transcriptionally and translational levels [2]. Increasing numbers of studies have indicated that lncRNAs play key roles in tumorigenesis and may be used in the diagnosis of cancers [3].

LncRNA microarray involving 10 gastric cancer tissues and 2 non-tumorous gastric tissues was performed by our team previously (GSE72307). Linc01133 was among the most differentially expressed lncRNAs. Linc01133 locates in chromosome 1q23.2 and is deregulated in a wide range of tumors. It was first identified as an oncogene in lung squamous carcinoma [4]. However, accumulating studies suggested that the function of linc01133 is multifaced and tissue-specific [5]. It was reported that linc01133 plays oncogenic roles in pancreatic cancer, hepatocellular carcinoma, renal cell carcinoma, endometrial carcinoma and cervical squamous carcinoma, while in colorectal carcinoma, linc01133 inhibits the malignant progression [5, 6]. However, it is not well understood how linc01133 regulate the growth of gastric cancer cells.

In the present study, we found that linc01133 was significantly upregulated in gastric cancer tissues compared to non-tumorous gastric tissues. We show that linc01133 functions as an endogenous competing RNA of miR-145-5p to upregulate the expression of YES1. Linc01133 upregulated the expression of the key cell cycle regulators such as CDK4, CDK6 and cyclin D1 by enhancing YES1-dependent YAP1 nuclear translocation to promote cell cycle progression.

## Results

### Linc01133 is upregulated in gastric cancer tissues

**Fig. 1:**
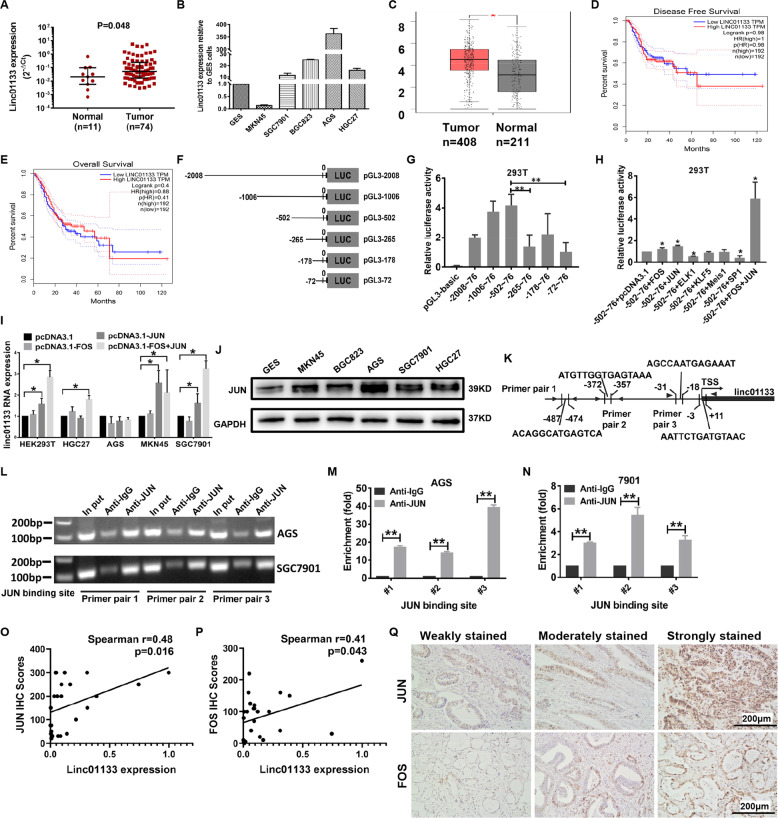
Relative expression of linc01133 in gastric cancer tissue. **A** RT-qPCR showed that linc01133 expression was upregulated in gastric cancer tissues compared to that in the normal gastric tissues (Mann–Whitney test, *p* = 0.048). **B** The expression of linc01133 was upregulated in gastric cancer cells compared to the immortalized normal gastric epithelial cells. **C** Online tool GEPIA analysis of linc01133 expression combining the TCGA and GTEx database. **D**, **E** GEPIA analysis of disease-free survival and overall survival of gastric patients with high (*n* = 192) and low (*n* = 192) linc01133 expression. **F** Schematic illustration of the luciferase vectors containing linc01133 promoter regions. **G** Luciferase assay confined the core regulatory regions to −502~−265 bp and −72~ +76 bp up-stream of transcriptional start site. **H** Luciferase assay showed that co-expression of c-JUN and c-FOS significantly increased the pGL3-502 luciferase activity. **I** Co-expression of c-JUN and c-FOS enhanced linc01133 expression in HEK293T, HGC27, MKN45 and SGC7901 cells but not in AGS cells. **J** JUN expression in gastric cancer cell lines. **K** Schematic illustration of the c-JUN-binding sites and the sites of primer pair design for ChIP assay. **L–N** ChIP-qPCR analyses showed that the promoter amplicons in the JUN-binding sites were significantly enriched by anti-Jun antibody. **O**, **P** The expression of Linc01133 was positively correlated with that of c-JUN and c-FOS in gastric cancer tissues (*n* = 25). **Q** Representative staining patterns of c-Jun and c-FOS detected by immunohistochemistry in gastric cancer tissues. Each experiment was performed in triplicates. Data are shown as mean ± SD, *P < 0.05, **P < 0.01.

Previously, lncRNA microarray was performed on 10 gastric cancer tissues and 2 normal gastric tissues by our team (GSE72307). Linc01133 was among the most differentially expressed lncRNAs. We subsequently confirmed the upregulation of linc01133 by 2.54-fold (median change) in gastric cancer tissues compared to normal gastric tissues (*P* = 0.048, Fig. 1A). The relationship between linc01133 expression and clinicopathological characteristics was also analyzed (Table 1). The expression of linc01133 was positively related with tumor size (*p* = 0.03) and differentiation (*p* = 0.003). Furthermore, linc01133 expression was significantly increased in four out of the five gastric cancer cell lines examined compared to the immortalized normal gastric epithelial cell GES-1 (Fig. 1B). An online tool GEPIA combining the TCGA data and GTEx data was utilized to further analyze the expression of linc01133 in gastric cancer tissue and matched normal tissue. Similar as we had found, linc01133 were significantly upregulated in gastric cancers compared to that in the normal gastric tissues, but having little impact on the outcome of the overall survival or the disease-free survival of gastric cancer patients (Fig. 1C–E).

**Table 1:** Association between linc01133 expression and clinicopathologic characteristics in gastric cancer patients.

Variables	Cases	linc01133 expression		*p* value
		Low	High	
Age				
≤60	25	12	13	0.38
>60	44	21	23	
Missing	5	4	1	
Gender				
Female	10	6	4	0.08
Male	60	27	33	
Missing	4	4	0	
Tumor size				
≤4 cm	18	13	5	0.03
>4 cm	53	22	31	
Missing	3	2	1	
LNM				
N0	21	12	9	0.28
N1-N3	47	20	27	
Missing	6	5	1	
Differentiation				
Well	4	4	0	0.003
Moderate	16	7	9	
Poor	47	19	28	
Missing	7	7	0	
Depth of invasion				
Mucous layer	1	1	0	0.14
Muscular layer	9	7	2	
Serosal layer	58	25	33	
Missing	6	4	2	

### The expression of linc01133 is driven collaboratively by c-FOS and c-Jun

To figure out the up-stream regulation of linc01133, 6 fragments spanning from −2000 bp up-stream to +76 bp down-stream of the transcriptional starting sites (TSS) of the linc01133 were constructed to pGL3-basic vector (Fig. 1F). All the six constructs displayed high luciferase activity compared to pGL3-basic control vector. However, obvious declines in luciferase activity were observed between pGL3-502 and pGL3-256 constructs, pGL3-72 and pGL3-basic constructs respectively, indicating important regulatory role of these two regions (Fig. 1G). Online tool PROMO and JASPAR predicted that transcription factor SP1, KLF5, ELK1, Meis1, JUN and FOS could potentially bind to the two regions with high scores. Over-expression of c-JUN or c-FOS alone caused mild increase in luciferase activity of pGL3-502~76 construct. As c-JUN and c-FOS form heterodimers to exert their function, we confirmed that simultaneous overexpression of c-FOS and c-JUN resulted in prompt increase in promoter activity in HEK293T cells (Fig. 1H). FOS and JUN overexpression increased linc01133 mRNA level in MKN45, SGC7901 and HGC27 gastric cancer cells, but not in AGS cells (Fig. 1I). We speculated it might be due to the higher endogenous expression of both JUN and linc01133 in AGS cells (Fig. 1B, J).

There were 4 predicted JUN-binding sites in the linc01133 promoter region. Two of them located between −502 and −256 bp up-stream of TSS. The other two located very close to each other between −72 and +76 bp (Fig. 1K). To verify the direct binding of JUN to linc01133 promoter. Chromatin-immunoprecipitation assay was conducted in AGS and SGC7901 gastric cancer cells. 3 pairs of primers were designed flanking the predicted binding sites for the subsequent qPCR validation (Fig. 1K). Fragments with JUN-binding sites were significantly precipitated by the anti-JUN antibody compared to the IgG antibody in both cell lines. Of note, AGS cells with higher endogenous linc01133 expression enriched much more JUN-binding sites-containing promoter amplicons compared to SGC7901 cells with low endogenous linc01133 expression (Fig. 1L–N).

Furthermore, we detected the expression of c-JUN and c-FOS by immunohistochemistry (IHC) on human gastric cancer tissues. Positive correlations were found between the IHC scores of c-JUN or c-FOS and linc01133 expression level (Fig. 1O–Q). Taken together, our findings demonstrate that the expression of linc01133 is driven collaboratively by c-FOS and c-Jun.

### Linc01133 promotes proliferation and cell cycle transition of gastric cancer cells

**Fig. 2:**
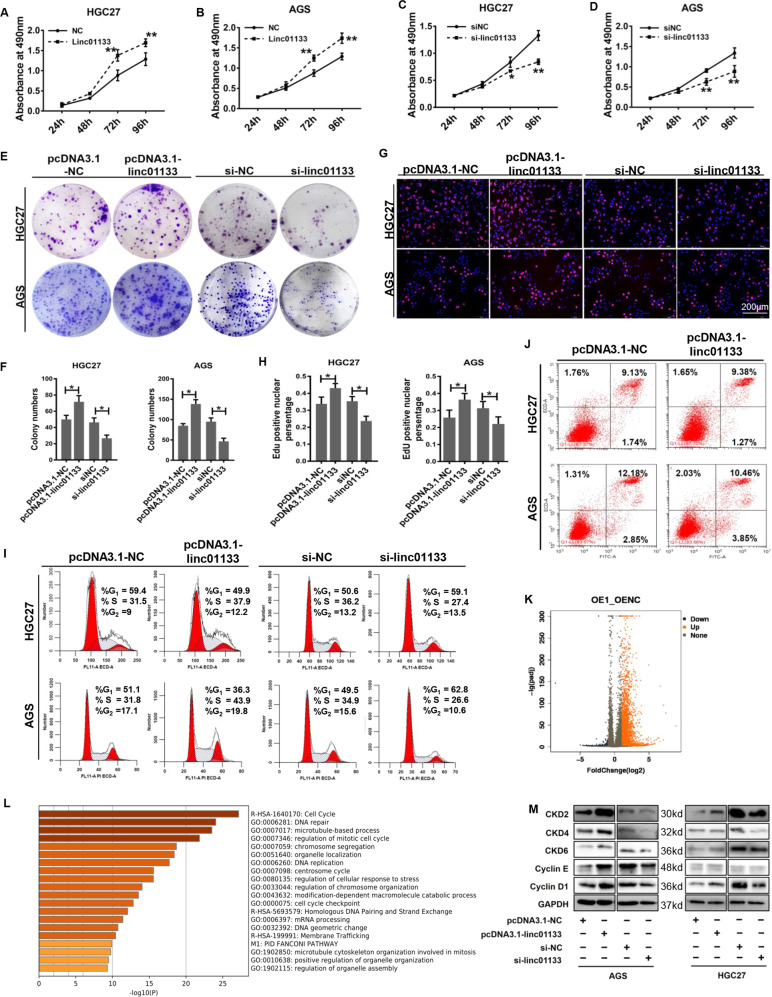
Linc01133 promotes the growth and cell-cycle transition of tumor cells in vitro. **A–D** Overexpression of linc01133 increased while knocking-down linc01133 decreased the cell viability by MTS assay. **E**, **F** Colony formation assays shows increased and decreased numbers of colonies formed by linc01133 manipulation. **G**, **H** EdU assay shows the increased and decreased proliferating cells upon linc01133 overexpression or knocking-down. **I** Flow cytometry shows that linc01133 greatly increased the S phase cells and decreased the G0/G1 phase cells. **J** linc01133 has no impact on cell apoptosis. **K** Volcano plot shows the up and downregulated genes upon linc01133 overexpression by RNA-seq. **L** Gene ontology analysis using online tool Metascape shows that cell cycle and cell cycle checkpoint pathways were enriched. **M** Western blots show that linc01133 increased the expression of G1 to S phase transition related genes. Each experiment was performed in triplicates. Data are shown as mean ± SD, *P < 0.05, **P < 0.01.

Linc01133 was reported to be multifaced with different functions and expression patterns in different malignancies. To study the function of linc01133, we overexpressed or knocked down linc01133 in AGS and HGC27 gastric cancer cell lines. Over-expression of linc01133 greatly enhanced, while knocking-down of linc01133 hindered, the proliferation and colony formation potency of the cancer cells (Fig. 2A–F). The percentages of EdU positive cells were in good accordance with the expression state of linc01133 (Fig. 2G, H). Next, we examined the impact of overexpression or knockdown linc01133 on cell cycle transition and apoptosis. Our results indicated that overexpression of linc01133 significantly increased the cell numbers in the S phase and decreased the cells in the G0/G1 phase. Si-linc01133 had an opposite effect (Fig. 2I). Linc01133 had little impact on the apoptosis (Fig. 2J).

Moreover, HGC27 cells were transfected with pcDNA3.1-control vector or pcDNA3.1-linc01133 vector and subjected to mRNA-seq (GSE191227). 1882 genes were upregulated and 432 genes were downregulated more than 2 folds (Fig. 2K). GO analysis of the differentially expressed genes was performed using an online tool Metascape. Cell cycle was the most enriched biological process (Fig. 2L). We examined some key cell cycle regulators. The data showed that the expression of CDK2, CDK4, CDK6 and cyclin D1 were positively correlated with linc01133 in both AGS and HGC27 cells (Fig. 2M). Collectively, these findings indicated that linc01133 promoted proliferation and cell cycle transition of gastric cancer cells.

### Linc01133 functions as ceRNA of miR-145-5p

**Fig. 3:**
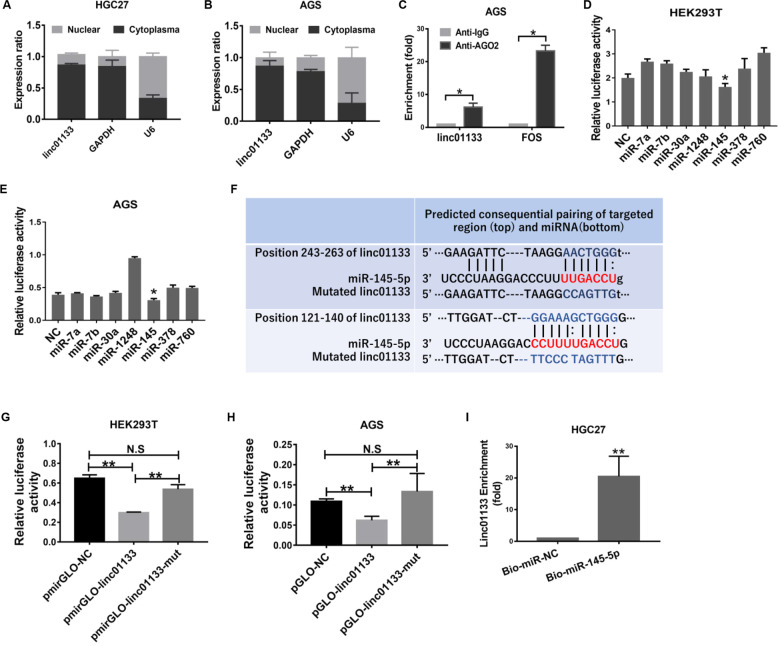
Linc01133 binds competitively to miR-145-5p. **A**, **B** Cytoplasmic and nuclear RNA isolation and RT-qPCR showed that linc01133 was predominantly localized in the cytoplasm in HGC27 and AGS cells. **C** RIP assay using anti-AGO2 antibody shows that linc01133 could be precipitated by anti-AGO2 antibody. **D**, **E** Out of the 7 candidate miRNAs, miR-145-5p reduced the luciferase activity of pmirGLO-linc01133 in HEK293T and AGS cells. **F** Predicted miR-145-5p binding sites and mutated sequence in linc01133. **G**, **H** MiR-145-5p reduced the luciferase activity of pmirGLO-linc01133 but not that with mutated miR-145-5p binding sites. **I** Biotin-labeled miR-145-5p pull-down assay showed enrichment of linc01133 by miR-145-5p.

Next, we explored the underlying mechanisms that linc01133 regulate cell cycle progression. We determined the localization of linc01133 by isolating cytoplasm and nuclear RNA separately. Linc01133 was predominantly cytoplasmic and AGO2 bounded, which was in accordance with previous reports (Fig. 3A–C). We assumed that linc01133 might exert its function as a competitive endogenous RNA.

RegRNA (http://regrna2.mbc.nctu.edu.tw/) predicated putative miRNAs potentially interacting with linc01133. miR-7a, miR-7b, miR-30a, miR-1248, miR-145-5p, miR-378 and miR-760, fitting in the following criteria: 1) predicted minimum free energy less than −15, 2) predicted interacting score more than 140 and 3) having been reported to be tumor-suppressive, were selected for further analysis. Luciferase assays showed that miR-145-5p reduced the luciferase activity of pmirGLO-linc01133 in HEK293T cell and gastric cancer cell AGS (Fig. 3D, E). RegRNA predicted 2 miR-145-5p binding sites in linc01133 (Fig. 3F). when both the binding sites were mutated, no significant reduction of luciferase activity was observed (Fig. 3G, H). Additionally, miR-145-5p and scrambled control miRNA were labeled with biotin at the 3’ end, and miRNA pull-down assay was carried out to detect whether miR-145-5p binds directly to linc01133. The same as we expected, linc01133 was enriched more than 20 folds by bio-miR-145-5p compared to bio-miR-NC (Fig. 3I).

**Fig. 4:**
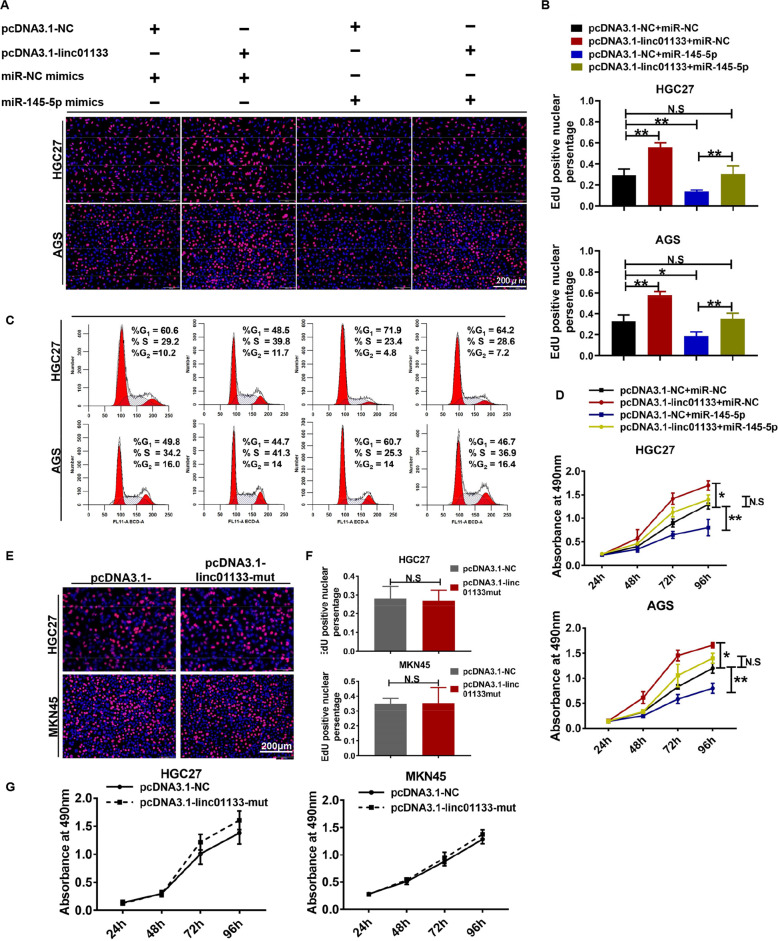
Linc01133 functions as a ceRNA of miR-145-5p. miR-145-5p counteracted with linc01133 in the proliferation and cell cycle progression of gastric cnacer cells demonstrated by EdU (**A, B**), FASC (**C**) and MTS assay (**D**). Linc01133 with miR-145-5p binding sites mutation failed to accelerate the tumor cell growth demonstrated by EdU (**E, F**) and MTS (**G**). Each experiment was performed in triplicates. Data are shown as mean ± SD, *P < 0.05, **P < 0.01, N.S no significance.

To answer the question that whether miR-145-5p could functionally counteract linc01133, we performed rescue experiments. HGC27 and AGS cells were transfected with linc01133 over-expression vector, miR-145-5p mimics or both. Linc01133 induced accelerating proliferation and S phase accumulation of gastric cancer cells, which were greatly hindered by miR-145-5p mimics demonstrating by EdU, MTS and FCSA assay (Fig. 4A–D). Moreover, linc01133 with mutated miR-145-5p binding sites failed to promote cell proliferation (Fig. 4E–G). Taken together, linc01133 and miR-145-5p functionally counteract with each other by direct binding.

### YES1 is a common target of miR-145-5p and linc01133

**Fig. 5:**
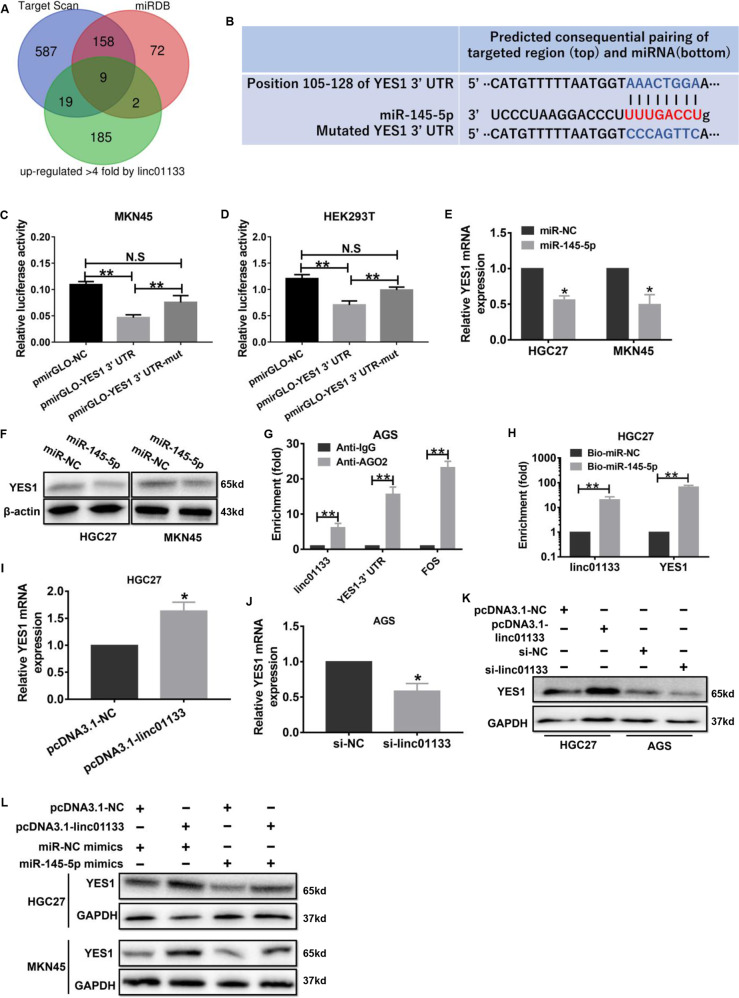
YES1 is a common target of miR-145-5p and linc01133. **A** Venn diagram depicting 9 candidate common targets of miR-145-5p and linc01133. **B** The complementary sequence of YES1 3’UTR with miR-145-5p seed sequence predicted by TargetScan and the mutated sequence. MiR-145-5p reduced the luciferase activity of pmirGLO-YES1 3’UTR but not that with miR-145-5p binding site mutation in MKN45 (**C**) and HEK293 cells (**D**). MiR-145-5p inhibited YES1 expression in both mRNA (**E**)and protein level (**F**). **G** YES1 3’UTR is AGO2 bounded demonstrated by RIP assay. **H** YES1 could be pulled down by biotin-labeled miR-145-5p. Linc01133 overexpression in HGC27 cells or knocking-down in AGS cells increased or decreased the expression of YES1 both in mRNA (**I, J**) and protein level (**K**). **L** Linc01133 greatly attenuated miR-145-5p induced YES1 down-regulation. Each experiment was performed in triplicates. Data are shown as mean ± SD, *P < 0.05, **P < 0.01.

To find the common target of miR-145-5p and linc01133, potential miR-145-5p targets predicted by TargetScan (www.targetscan.org) and miRDB (www.mirdb.org) were combined with the genes that upregulated more than 4 folds after linc01133 overexpression in the RNA-seq data. A list of 9 candidate genes, GMFB, UBE2W, IPMK, YES1, ZBTB33, UBA6, ZBTB10, SLC25A36 and PPP4R2, were generated (Fig. 5A). Among the 9 genes, YES1, a member of SRC kinase family, is reported to be upregulated in gastric cancer [7], and YES1 over-expression promoted gastric cancer cell growth in vitro [8]. In our RNA-seq data, YES1 was upregulated by more than 5 folds upon linc01133 overexpression. There is one potential miR-145-5p binding site positioned from 105 to 128 in YES1 3’ UTR (Fig. 5B). The luciferase activity of HEK293T and MKN45 cells transfected with pmirGLO-YES1 3’ UTR, but not that with mutated miR-145-5p binding site (pmirGLO-YES1 3’ UTR mut), was greatly reduced by miR-145-5p mimics (Fig. 5C, D). MiR-145-5p suppressed YES1 expression in both mRNA and protein levels (Fig. 5E, F). Moreover, the same as linc01133, YES1 could also be readily detected by qRT-PCR from RNA immunoprecipitated by anti-AGO2 antibody or pulled-down by biotin-labeled miR-145-5p (Fig. 5G, H). Our data demonstrated that YES1 is a target of miR-145-5p.

Additionally, the regulation of YES1 by linc01133 was confirmed by qRT-PCR and western blot (Fig. 5I–K). When HGC27 and MKN45 cells were co-transfected with miR-145-5p mimics and linc01133 expression vector, the YES1 down-regulation by miR-145-5p mimics was greatly attenuated by linc01133 expression (Fig. 5L). Collectively, YES1 is a common target of miR-145-5p and linc01133.

### YES1 confers the function of linc01133 in vitro and in vivo

**Fig. 6:**
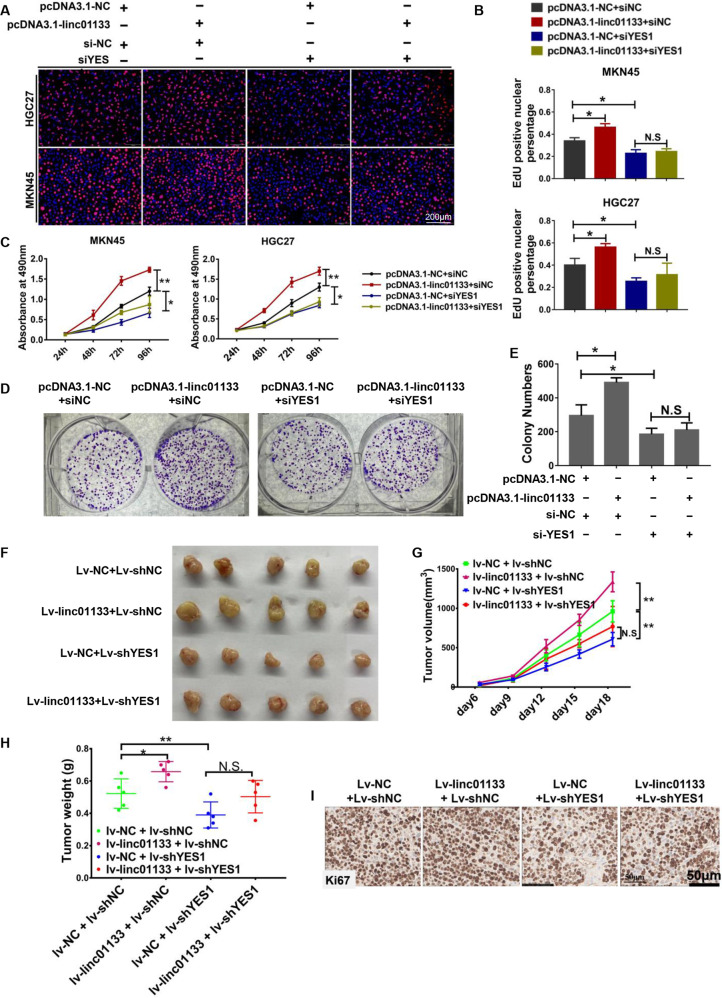
YES1 confers the function of linc01133 in vitro and in vivo. Knocking-down YES1 ameliorated linc01133 induced accelerating cell growth demonstrated by EdU assay (**A, B**) and MTS assay (**C**). **D–E** Colony formation assay showed decreased colony numbers formed by MKN45 cells co-transfected with pcDNA3.1-linc01133 and si-YES1. **F–H** The sizes, growth curves and weights of tumor xenografts showed that over-expression of linc01133 significantly enhanced tumor growth while simultaneous YES1 knocking-down inhibited tumor growth. **I** Ki-67 immuno-staining showed that knocking-down YES1 ameliorated linc01133 induced Ki-67 positive cells. Data are shown as mean ± SD, *P < 0.05, **P < 0.01, N.S no significance.

Since YES1 is a target of both linc01133 and miRNA-145-5p, we asked whether linc01133 exerts its function via up-regulating YES1. Indeed, YES1 knockdown ameliorated linc01133 induced accelerating cell growth completely as demonstrated by EdU and MTS assay (Fig. 6A–C). Colony formation assays demonstrated decreased colonies formed by MKN45 cells co-transfected with linc01133 expression vector and siRNA targeting YES1 (Fig. 6D, E).

To further study the function of linc01133 in gastric cancer cells in an in vivo system, we transfected MKN45 cells with Lv-linc01133 (expressing GFP), Lv-shYES1(expressing RFP) or both to generate cells with stable linc01133 overexpression, YES1 knockdown or cells simultaneously overexpressing linc01133 and knocking-down YES1. The generated cells were then subcutaneously injected into the nude mice (*n* = 5 for each group). Linc01133-overexpression greatly enhanced the xenograft tumor growth compared to the control group (tumor weight: *p* = 0.0397; tumor volume: *p* = 0.005), while the growth of YES1-knocked-down xenograft tumors was obviously impeded (tumor weight: *p* = 0.0317; tumor volume: *p* = 0.004). Moreover, YES1 ablation greatly hindered linc01133-induced tumor growth (Fig. 6F–I). Taken together, YES1 confers the function of linc01133 both in vitro and in vivo.

### YES1 confers the function of linc01133 via promoting YAP1 nuclear translocation

**Fig. 7:**
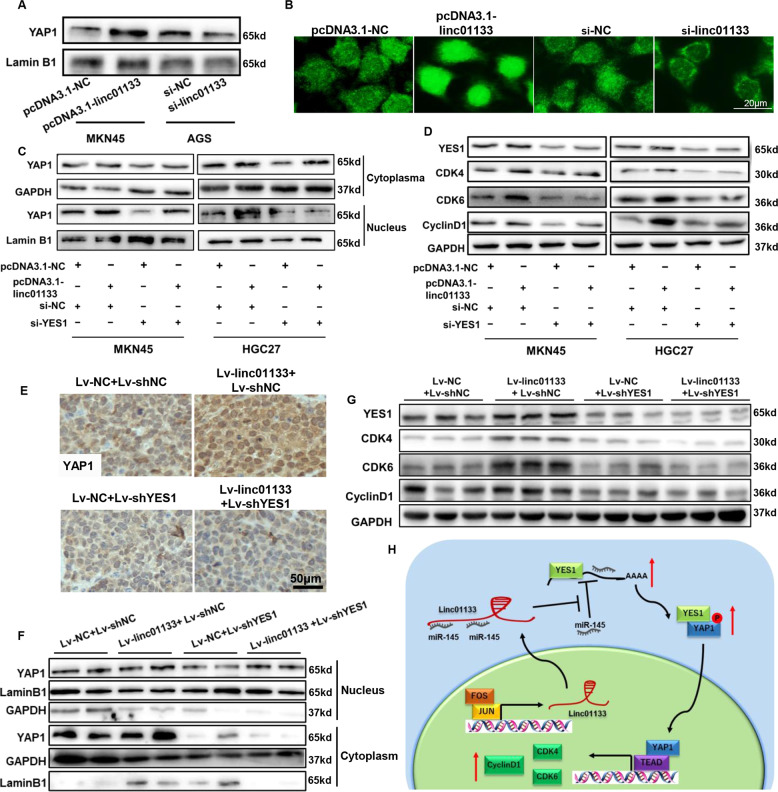
YES1 confers the function of linc01133 via promoting YAP1 nuclear translocation. **A**, **B** Western and fluorescence immuno-staining showed that over-expressing linc01133 increased while knocking-down linc01133 decreased nuclear YAP1. **C** Simultaneous YES1 knocking-down ameliorated linc01133 induced YAP1 nuclear accumulation in MKN45 and HGC27 cells. **D** Western blotting showed that CDK4, CDK6 and cyclin D1 expression was in good accordance with nuclear YAP1 accumulation. **E**, **F** Immunohistochemistry staining and western blotting showed YAP1 nuclear accumulation in tumor xenografts. **G** Western blotting for key regulators in G1/S phase transition in tumor xenografts. Each experiment was performed in triplicates. **H**. Schematic illustration of the mechanisms that linc01133 promotes the growth of gastric cancer cells.

YES1 belongs to the Src family of protein-tyrosine kinases. It is reported that YES1 binds and phosphorylates YAP1 (YES-associated protein 1), as a result protecting the latter from degradation. YAP1 then translocates to the nucleus and activates YAP–TEAD-dependent transcription [9]. We confirmed the up- and down-regulation of nucleus YAP1 by linc01133 overexpression or knockdown respectively in MKN45 and AGS gastric cancer cell lines (Fig. 7A, B). In accordance with previous reports, YES1 knockdown leads to reduced YAP1 accumulation both in the cytoplasm and in the nucleus (Fig. 7C). Interestingly, linc01133 induced YAP1 nuclear accumulation was largely lost upon YES1 knockdown, suggesting that YES1 was indispensable for linc01133 to upregulate YAP1 in the nucleus. Moreover, in the mouse tumor xenografts, linc01133 induced increased YAP1 expression both in the cytoplasm and in the nucleus, which were greatly ablated by simultaneous YES1 knockdown (Fig. 7E, F). YAP1 is a transcription co-activator regulating cell proliferation via enhancing the expression of cyclins and cyclin-dependent kinases (CDKs) [10, 11]. Indeed, the expression of CDK4, CDK6 and cyclin D1, which are expressed predominantly in the G1 phase and promotes the transition to S phase of the cell cycle, closely correlated with the expression of YES1 and YAP1 in gastric cancer cells and tumor xenografts (Fig. 7D, G).

Collectively, our data indicated that the function of linc01133 was conferred by YES1-dependent YAP1 nuclear accumulation.

## Discussion

Linc01133 is found to be aberrantly expressed in a variety of malignancies with controversial functions. In this study, we found that linc01133 was upregulated combining our microarray data, clinical gastric cancer samples validation and TCGA data bioinformatic analysis. Functional assays were performed to show that linc01133 accelerated the gastric cancer cell proliferation by promoting cell cycle transition from G1 to S phase. It was reported that linc01133 could promotes cell proliferation in pancreatic ductal adenocarcinoma through upregulation of cyclin G1 [12]. We found that in gastric cancer cells CDK4, CDK6 and cyclinD1 were increased upon linc01133 overexpression. We speculated that the accelerated proliferation induced by linc01133 was due to the increased CDK4, CDK6 and Cyclin D1 levels.

Linc01133 was a cytoplasm-located, AGO2-bounded RNA, suggesting a possible role as ceRNA. Indeed, several studies have reported that linc01133 functions as a ceRNA for several miRNAs in different cancer types. Linc01133 could sponge miR-422 in osteosarcoma and miR-30b-5p in renal cell carcinoma to promote malignant behaviors of tumor cells [6, 13]. Next, we proved that linc01133 could bind to miR-145-5p competitively to relief the repression to YES1 by the latter. MiR-145-5p is a tumor-suppressor which is downregulated in a wide range of malignant tumors. miR-145 inhibits tumor cell proliferation, invasion, and metastasis [14]. We provided direct evidence that linc01133 interacting with miR-145-5p via biotin-labeled miR-145-5p pull-down assay followed by RT-qPCR examination. Furthermore, functional rescue assay was carried out to show that linc01133 and miR-145-5p mutually counteracted with each other functionally.

Combining RNA-seq data and online target prediction, YES1, which is a potential miR-145-5p target, was found to be upregulated by linc01133. We proved that YES1 was a target of miR-145-5p in gastric cancer cells using luciferase assay and biotin-labeled miR-145-5p pull-down assay. YES1, a member of SRC non-receptor tyrosine kinase family, is a well-known oncogene. We demonstrated that YES1 confers the oncogenic activity of linc01133 both in vivo and in vitro. YES-associated protein 1(YAP1) is the key effector of Hippo signaling pathway. Upon activation, YAP1 translocated to the nucleus where it exerts its function as a transcriptional activator to initiate the expression of a variety of genes including CDK6, cyclinD1 [10, 11]. It is reported that YES1 promotes YAP1 nuclear accummulation by tyrosine phosphorylation of YAP1 and inhibiting its nuclear export [9, 15–17]. We demonstrated that overexpression linc01133 significantly increased YAP1 nuclear accumulation with a simultaneous elevation in CDK4, CDK6 and cyclin D1 levels. Knock-down of YES1 attenuated the linc01133 induced YAP1 nuclear translocation, suggesting a pivotal role of YAP1 in linc01133 induced cell cycle transition.

In pancreatic cancer, the expression of linc0113 is regulated by C/EBP beta. It is not clear how linc01133 is regulated in gastric cancer. For the first time, we identified JUN together with FOS as the regulators of linc01133 by means of luciferase assay and ChIP assay. Simultaneous overexpression of JUN and FOS leads to stably increased linc01133 expression in MKN45, HGC27 and SGC7901 but not in AGS gastric cancer cells. We inferred that may due to the high endogenous linc01133 expression in AGS cells.

In conclusion, linc01133, a JUN and FOS regulated lncRNA, promoted gastric cancer cell growth as a ceRNA for miR-145-5p via YES1 mediated YAP1 nuclear translocation.

## Materials and methods

### Human GC samples

A total of 74 cases of GC samples (22 cases from Qilu hospital affiliated to Shandong University, 40 cases from Shandong Provincial Hospital and 12 cases from Weifang Hospital) underwent gastrectomy from the year 2013-2016 were collected. Pathological classification and tumor staging were performed according to the criteria established by the World Health Organization (WHO) (2010) and UICC/AJCC TNM classification (the 7^th^ edition). Informed consents were obtained from all the recruited patients. The study was approved by the Ethics Committee of Shandong University and conducted in accordance with the ethical guidelines of the World Medical Association Declaration of Helsinki.

### Immunohistochemical staining (IHC)

IHC was performed on paraffin-embedded sections of human gastric cancer tissues or tumor xenografts of nude mice using antibodies against c-JUN (1:50, ab40766, Abcam,), c-FOS (1:1000, ab222699, Abcam), Ki-67 (1:100, 27309-1-AP, Proteintech, Wuhan, China) and YAP1 (1:100, 13584-1-AP, Proteintech, Wuhan, China). The staining intensity and percentage of positive nucleuses was evaluated by two professional pathologists independently. The staining intensity was assessed as 0 (Negatively stained), 1 (weakly stained), 2 (moderately stained) and 3 (strongly stained). The IHC scores were calculated according to the formula: IHC score = P1 × 1 + P2 × 2 + P3 × 3 (P: percentage of positive nucleuses).

### GC cell lines and culture conditions

Gastric cancer cell lines MKN45, SGC-7901, HGC-27 and AGS were obtained from the Cell Bank of Chinese Academy of Sciences and cultured in RPMI-1640 medium supplemented with 10% fetal bovine serum (Hyclone). HEK293T cells were cultured in DMEM (Hyclone) supplemented with 10% fetal bovine serum. All cell lines were maintained in a humidified cell incubator at 37 °C with an atmosphere of 5% CO2.

### Plasmid construction and, siRNA and miRNA mimics

The expression vectors of linc01133, ELK1, KLF5, Meis1, SP1, JUN and FOS were generated by inserting the full length or the CDS of each gene into pcDNA3.1(+). The putative linc01133 promoter regions were PCR-amplified from the genomic DNA of MKN45 cells and inserted into NheI/HindIII site of the pGL3-basic vector. Full length of linc01133 with mutated miR-145-5p binding sites, YES1 3’ UTR and YES1 3’UTR with mutated miR-145-5p binding site were synthesized by BioSune (Jinan, China) and inserted into pmirGLO SacI/XbaI site. LV-linc01133-GFP-puromycine were constructed by Genepharma (Shanghai, China). LV-shYES1-RFP-Blasticidin were constructed by Vigene (Vigene biosciences, Jinan, China).

2 siRNA targeting linc01133 and 3 siRNA targeting YES1 were designed by Genepharma (Shanghai, China). The ones with the highest knockdown efficacy were selected for the following experiments. MicroRNA mimics were synthesized by Genepharma (Shanghai, China). All the primer and siRNA sequences can be found in Supplementary Table 1.

### Cell transfection

MKN45, AGS or HGC27 cells were transfected with Turbofect transfection reagent (Thermo) or X-tremeGENE transfection reagent (Roche) for plasmid or siRNA transfection respectively. Co-transfection of plasmid DNA and siRNA (or miRNA mimics) were performed by Lipo3000 Transfection Reagent (Invitrogene).

### RNA extraction and RT-qPCR

Total RNA was extracted using TRIzol® reagent (Invitrogen) according to the manufacturer’s manual. cDNA was synthesized from 1 μg of the total RNA with a ReverTra Ace qPCR RT kit (Toyobo). Real-time quantitative PCR was then performed using SYBR Green Real-time PCR Master Mix (Roche) and an Applied Roche LightCycler® 96 instrument. Relative expression was normalized to GAPDH expression, which yielded a 2^−ΔCt^ value. Primer sequences were shown in Supplementary Table 1.

### Western blotting

The following primary antibodies were used in this study: CDK4 (1: 1000; CST), CyclinD1 (1: 1000; CST), CDK6 (1: 1000; CST), YAP1 (1: 500; Santa Cruz,), YES1 (1: 500; Proteintech), and GAPDH (1: 5000; Proteintec). Independent experiments for western blotting were performed at least thrice.

### Cell proliferation assays and colony formation assay

Cell Titer 96 nonradioactive cell proliferation (MTS) (Promega BioSciences, Madison, WI, USA), CellLight™ EdU cell proliferation detection (EdU) (RiboBio), and colony formation assays were performed to test the proliferation ability of the GC cells following the manufacturer’s protocols. For the MTS assay, cell proliferation was measured at 24, 48, 72 and 96 h after transfection. For the colony formation assay, 800 cells were seeded into six-well plates. After culturing for 2 weeks, the colonies were fixed and stained, and then, the colonies were counted.

### Flow cytometry analysis of cell apoptosis and cell cycle

The GC cells were collected 48 h after transfection, and the cell cycle and apoptosis were evaluated using the Cell-cycle Detection Kit and FITC Annexin V Apoptosis Detection kit (BestBio, Shanghai, China) respectively. The harvested cells were then analyzed by flow cytometry (FACScan, BD Biosciences).

### Dual-luciferase reporter assay

To identify linc01133 interacting miRNAs, HEK293 cells or AGS gastric cancer cells were co-transfected with individual candidate miRNA and pmirGLO vectors containing full length of linc01133, YES1 3’UTR or their miR-145-5p binding site mutated counterparts. To determine the core regulatory promoter region, HEK293 cells was co-transfected with pGL3-promoter reporter plasmid, pRL-TK plasmid as an internal control and transcription factor overexpressing vector. 48 h after transfection, luciferase activities were detected using a Dual-Luciferase Reporter Assay System (Promega) according to the manufacturer’s instructions.

### Biotin-labeled miR-145-5p pull-down assay

Biotin-labeled miR-145-5p pull-down assay was performed as previously described [18]. HGC27 cells were transfected with 3’biotin-labeled miR-145-5p or a negative control miRNA (Genepharma) for 48 h. M-280 streptavidin magnetic beads (Invitrogen) were pre-incubated with 10 ul yeast tRNA for 2 h at 4 °C, and then incubated with the cell lysates overnight at 4 °C on a rotator. The bound RNAs were purified for further RT-qPCR analysis.

### Chromatin immunoprecipitation assay

Chromatin immunoprecipitation (ChIP) assay were performed using the EZ-Magna ChIP Chromatin Immunoprecipitation Kit (Millipore) according to the manufacturer’s instructions. For each reaction, 1ug anti-Jun antibody (Santa cruz) or anti-mouse IgG antibody was used to immnoprecipitate JUN-bounded DNA fragments. The co-precipitated DNA was quantified by qPCR. Primer sequences are shown in Supplementary Table 1.

### In vivo tumor formation assay

BALB/c nude mice (male, 4 weeks old) were purchased from Weitong lihua Biotechnology (Beijing, China). MKN45 cells were co-transfected with lv-NC+lv-shNC, lv-linc0113+lv-shNC, lv-NC+lv-shYES1 or lv-linc01133+lv-shYES1 respectively. Stably transfected cells were selected and maintained. 4 × 10^5^ stably transfected MKN45 cells were inoculated sub-cutaneously into the axillary fossa (*n* = 5 each, randomized allocated). Tumor growth was examined every three days and tumor volumes (V) were calculated as V = (tumor length × width^2^)/2. Four weeks after injection, the mice were sacrificed, and the tumors were isolated and snap-frozen for RNA and protein extraction. Tumor nodules were histological assessed by Hematoxylin and Eosin (HE) staining.

### Statistical analysis

Statistical analysis was performed using Graphpad Prism 7(GraphPad Software). Significant differences were confirmed using Student’s *t* test for two groups or oneway ANOVA for three groups. Each experiment was repeated in triplicates. *P* < 0.05 was considered statistically significant.

## Supplementary information

Supplementary table1

consent to author list change

checklist

## Data Availability

The datasets used and analyzed during the current study are available from the corresponding author on reasonable request.
