# Supplementary material for: Linc01133 contributes to gastric cancer growth by enhancing YES1-dependent YAP1 nuclear translocation via sponging miR-145-5p
Source: Cell Death Dis. 2022 Jan 11;13(1):51. doi: 10.1038/s41419-022-04500-w (PMC8752595; doi:10.1038/s41419-022-04500-w)
Supplement: Supplementary file 1 — Supplementary table1 [file 41419_2022_4500_MOESM1_ESM.docx]

**Supplementary table: Primer sequences and siRNA sequences used in this study**

| Primer sequences for detecting gene expression | | |
| --- | --- | --- |
| Linc01133 | Forward | TCCTAATCTCACCACAGCCT |
|  | Reverse | CACCACTGATGTCTACCATG |
| YES1 | Forward | CAGTGGTGCCAAGTTCAT ATC |
|  | Reverse | GATCTTGCTTCCCACCAATCTC |
| GAPDH | Forward | GCACCGTCAAGGCTGAGAAC |
|  | Reverse | TGGTGAAGACGCCAGTGGA87 |
| Primer sequences for amplifying the promoter region | | |
| -2008 Forward | | TGGCTAGCCTCACAGATGGCTAAG |
| -1006 Forward | | TGGCTAGCCTGTGTCCAAGTGTTCTC |
| -502 Forward | | TGGCTAGCTACAGGCATGAGTCACTGCC |
| -256 Forward | | TGGCTAGCGTCCACAACGGTAATCAACT |
| -178 Forward | | TGGCTACGAGAAAGACGTCCTCCAC |
| -72 Forward | | TGGCTAGCGACTTTCTCTCTCTTCTTCC |
| +76 Reverse | | CGAAGCTTCCATTCTCTCCACCACAG |
| Primer Sequences in ChIP assay | | |
| Primer Pair #1 | Forward | TCCCGACCTCGTGATCCAC |
|  | Reverse | GTGGCCTTCCACTGTCATAG |
| Primer Pair #2 | Forward | CAACTGGGAAGTATGCAG |
|  | Reverse | CAGAATTGCTCATGGAATAG |
| Primer Pair #3 | Forward | CTTCCTGGATCTTTAGGAG |
|  | Reverse | CAGCTTTTCACTGCAACC |
| siRNA targeting sequences | | |
| Si-Linc01133 | GAGUAGAAGACAGUAUCAAGA | |
| Si-YES1 | GAAGCAAGAUCAAUCGCUAdTdT | |
