# Supplementary material for: Linc01133 contributes to gastric cancer growth by enhancing YES1-dependent YAP1 nuclear translocation via sponging miR-145-5p
Source: Cell Death Dis. 2022 Jan 11;13(1):51. doi: 10.1038/s41419-022-04500-w (PMC8752595; doi:10.1038/s41419-022-04500-w)
Supplement: Supplementary file 2 — consent to author list change [file 41419_2022_4500_MOESM2_ESM.pdf]

Re: Autho list change

昨天晚上9:57

Dear Professor Gao，

I agree to the anthor list change.
Thank you！

Yujing Sun

**Yujing Sun**

邮箱： candysyj4@163.com

---- Replied Message ----

From 高鹏<gaopeng@sdu.edu.cn>
Date 12/08/2021 21:51
To yujing sun<candysyj4@163.com>
Subject Autho list change

Dear Dr.Sun,

For the article "Linc01133 contributes to gastric cancer growth by enhancing YES1-dependent YAP1

---

回复： Autho list change

昨天晚上10:10

Dear Professor Gao,
Thank you for the information about the change of authors. I agree with adding Wen-jie Zhu to the list of authors.
Best wishes and good night!
Tian Yuan

------------------ 原始邮件 ------------------

**发件人:** "高鹏"<gaopeng@sdu.edu.cn>;
**发送时间:** 2021年12月8日(星期三) 晚上9:53
**收件人:** "Helium"<2965188722@qq.com>;
**主题:** Autho list change

Dear Dr.Tian,

For the article "Linc01133 contributes to gastric cancer growth by enhancing YES1-dependent YAP1 nuclear translocation via sponging miR-145-5p"， which We have submitted to the journal "Cell death and disease",

we added Wen-jie Zhu as the new co-author.

The new author list is :

Yujing Sun, Yuan Tian, Junyi He, Yaru Tian, Guohao

---

回复： Autho list change

昨天晚上10:09

I agree to this change.
发自我的华为手机

-------- 原始邮件 --------
发件人： 高鹏 <gaopeng@sdu.edu.cn>
日期： 2021年12月8日周三 晚上9:54
收件人： hjy_008@126.com
主 题： Autho list change

Dear Dr.He,

For the article "Linc01133 contributes to gastric cancer growth by enhancing YES1-dependent YAP1 nuclear translocation via sponging miR-145-5p"， which We have submitted to the journal "Cell death and disease",

we added Wen-jie Zhu as the new co-author.

The new author list is :

Yujing Sun, Yuan Tian, Junyi He, Yaru Tian, Guohao Zhang, Ruinan Zhao, Wen-jie Zhu, Peng Gao

Please reply to this email to confirm your agreement to this change.

---

Re: Autho list change

昨天晚上10:02

Dear Dr.Gao,

I confirm that I agree to the author list change.

Sincerely!
Yaru Tian

On 12/8/2021 21:53，高鹏<gaopeng@sdu.edu.cn> wrote：

Dear Dr.Tian,

For the article "Linc01133 contributes to gastric cancer growth by enhancing YES1-dependent YAP1 nuclear translocation via sponging miR-145-5p"， which We have submitted to the journal "Cell death and disease",

we added Wen-jie Zhu as the new co-author.

The new author list is :

Yujing Sun, Yuan Tian, Junyi He, Yaru Tian, Guohao

---

Re: Autho list change

昨天晚上10:05

Agree with the author listing order.

---Original---

**From:** "高鹏"<gaopeng@sdu.edu.cn>
**Date:** Wed, Dec 8, 2021 21:55 PM
**To:** "623092550"<623092550@qq.com>;
**Subject:** Autho list change

Dear Dr.Zhang,

For the article "Linc01133 contributes to gastric cancer growth by enhancing YES1-dependent YAP1 nuclear translocation via sponging miR-145-5p"， which We have submitted to the journal "Cell death and disease",

we added Wen-jie Zhu as the new co-author.

The new author list is :

Yujing Sun, Yuan Tian, Junyi He, Yaru Tian, Guohao Zhang, Ruinan Zhao, Wen-jie Zhu, Peng Gao

Please reply to this email to confirm your agreement to this change.

---

Re: Autho list change

昨天晚上10:09

I agree with this change

发自我的iPhone

------------------ Original ------------------

**From:** 高鹏 <gaopeng@sdu.edu.cn>
**Date:** Wed,Dec 8,2021 9:56 PM
**To:** 赵瑞男 <231648042@qq.com>
**Subject:** Re: Autho list change

Dear Dr.Zhao,

For the article "Linc01133 contributes to gastric cancer growth by enhancing YES1-dependent YAP1 nuclear translocation via sponging miR-145-5p"， which We have submitted to the journal "Cell death and disease",

we added Wen-jie Zhu as the new co-author.

The new author list is :

Yujing Sun, Yuan Tian, Junyi He, Yaru Tian, Guohao Zhang, Ruinan Zhao, Wen-jie Zhu, Peng Gao

---

Re: Autho list change

昨天晚上10:03

I agree to this change

**朱朱**

邮箱： 15266203531@163.com

签名由 网易邮箱大师 定制

On 12/08/2021 21:56, 高鹏 wrote:

Dear Dr.Zhu,

For the article "Linc01133 contributes to gastric cancer growth by enhancing YES1-dependent YAP1 nuclear translocation via sponging miR-145-5p"， which We have submitted to the journal "Cell death and disease",

we added Wen-jie Zhu as the new co-author.

The new author list is :

Yujing Sun, Yuan Tian, Junyi He, Yaru Tian, Guohao Zhang, Ruinan Zhao, Wen-jie Zhu, Peng Gao
